# Periodontal status of rheumatoid arthritis patients in khartoum state

**DOI:** 10.1186/1756-0500-4-460

**Published:** 2011-10-28

**Authors:** Safa K Abdelsalam, Nada T Hashim, Emitithal M Elsalamabi, Bakri G Gismalla

**Affiliations:** 1 Department of periodontology, School of Dentistry, Elneilein University, Khartoum, Sudan; 2 Department of Periodontology, Khartoum North Dental Teaching Hospital, Khatroum, Sudan; 3 Department of rheumatology, Faculty of Medicine, University of Medical Sciences and Technology, Khartoum, Sudan; 4 Department of Oral Rehabilitation, Faculty of Dentistry, University of Khartoum, Khartoum, Sudan

**Keywords:** periodontal health, Rheumatoid arthritis

## Abstract

**Background:**

Few studies have investigated the periodontal condition among Rheumatoid arthritis in Sudan. The present study described the periodontal condition among Sudanese patients suffering from rheumatoid arthritis and to compare them with those of non-rheumatic subjects.

**Methods:**

A group of eighty rheumatoid arthritis patients was selected from Patient's Rheumatoid Clinics in Khartoum State during the period of January to May 2010. A control group of eighty patients with the same age and gender was selected for the study. Both Rheumatoid arthritis patients and the control group were examined for their plaque index, gingival index, and clinical attachment loss.

**Results:**

The results revealed that there were no significant differences in plaque and gingival index among study and control groups, with mean plaque index of (1.25 ± 0.4) for patients and (1.17 ± 0.28) for the control group (p-value is 0.3597). The mean gingival index was (1.2 ± 0.24) for the patients and (1.2 ± 0.33) for the control (p = is 0.3049). The results showed statistically significant differences in clinical attachment loss between study and control groups, with mean clinical attachment loss of (1.03 ± 0.95) for the study group and (0.56 ± 0.63) for the control group (p = 0.0002). The study revealed that no association exists between the type of drug used to treat rheumatoid arthritis (NSAIDs & DMARDs) and the periodontal parameters (plaque index, gingival index, and clinical attachment loss).

**Conclusion:**

A significant relationship between periodontal disease and Rheumatoid Arthritis does exist, but no difference between plaque and gingival index has been detected among study and control groups.

## Background

The oral cavity is thought to be the window to the body because oral manifestations accompany many systemic diseases [1]. Periodontitis is a common disease worldwide that has a primary bacterial etiology and is characterized by dysregulation of the host inflammatory response which eventually results in soft and hard tissue destruction [2,3]

Rheumatoid arthritis (RA) is a chronic destructive inflammatory disease characterized by the accumulation and persistence of an inflammatory infiltrate in the synovial membrane that leads to synovitis and the destruction of the joint architecture [2].

Rheumatoid arthritis (RA) occurs worldwide with prevalence of 1% in the population, most common in females [4], affecting women three times more than men [5,6]. It is estimated that arthritis and other rheumatic conditions affect 42.7 million Americans [7] with prevalence of 0.5 to 1% in Western population [8].

While the etiology of these two diseases may differ, the underlying pathogenic mechanisms are remarkably similar and it is possible that individuals manifesting both periodontitis and RA may suffer from a unifying underlying systemic dysregulation of the inflammatory response [2]. There is almost universal acceptance that a variety of cytokines and matrix metalloproteinases (MMPs) are upregulated and intimately involved in the pathogenesis of both periodontitis and RA; many of these effector molecules appear to be common to both diseases [3]. High levels of proinflammatory cytokines, including IL-1b and tumor necrosis factor-alpha (TNF-a), and low levels of cytokines which suppress the immunoinflammatory response, such as IL-10 and transforming growth factor-b (TGF-b), have been detected in periodontitis as well as in Rheumatoid Arthritis [9].

Natural history studies of periodontal disease in humans indicate the presence of three distinct subpopulations: [10].

1) no progression of periodontal disease, in which around 10% of the population manifest very little or no disease which is of particular consequence to dentition; 2) moderate progression, affecting around 80% of the population and representing a very slowly progressing form of disease that generally can be easily managed via routine therapies; and 3) rapid progression, affecting approximately 8% of individuals whereby extensive periodontal destruction occurs which can be very difficult to control.

On the other hand, three types of disease manifestations can also be observed in RA populations:

1) Self-limited: in these cases, individuals originally presenting for RA have no evidence of disease 3 to 5 years later; [11].

2) Easily controlled: the disease is relatively easily controlled with only non-steroidal anti-inflammatory drugs (NSAIDs); [12]

3) Progressive: these patients generally require second-line drugs, which often still do not fully control the disease [13].

It must be recognized that periodontitis differs in one significant way from RA through our understanding that the subgingival biofilm is a key etiologic factor in periodontitis. Unlike periodontal disease, no specific bacterial etiology has been identified for RA. Thus, while host modifications of disease processes are possible for periodontitis, controlling the bacteria that cause periodontal infections remains a significant focus for periodontal treatment and prevention. Host modification can be only an adjunct treatment for periodontitis. However, until an etiologic factor can be found for RA, host modification remains the primary treatment [3].

Currently, the first line of treatment for RA is NSAIDs such as aspirin, naproxen, diclofenac, and ibuprofen. Their mechanism of action through the inhibition of Cyclooxygenase (COX) synthesis produces both analgesic and antipyretic properties. Although these medications are effective in reducing the pain symptoms in RA, they do not significantly alter its course [14].

The use of NSAIDs for the treatment of chronic periodontitis has been studied over the past 20 years [15]. While the results appear promising, the widespread clinical use of these medications to alter the course of periodontitis has not been universal. Their use for the management of periodontitis appears to be a ''rebound'' effect to baseline following a cessation of the medication [16].

With the discovery of two COX enzymes responsible for PGE2 production, designated COX-1 and COX-2, a variety of COX-2 inhibitors have been studied for their potential to stop or slow down bone resorption. One of the first COX-2 inhibitors developed, Tenidap, has been shown to inhibit not only cyclooxygenase and PGE2 production but also IL-1, IL-6, and TNF-a production. To date, the potential of COX-2 inhibitors to modify bone resorption in periodontitis have not been thoroughly studied [3].

In contrast to NSAIDS, which do not significantly alter the course of RA, a newer family of medications designated as disease-modifying anti-rheumatic drugs (DMARDs) has been developed. This medication has demonstrated an ability to change the course of RA for at least one year as evidenced by sustained improvement in function, decreased synovitis, and prevention of further joint damage [17]. Examples of these medications include parenteral gold salts, methotrexate, sulfasalazine, hydroxychloroquine (antimalarial drug), penicillamine, azathioprine, and leflunomide. A major drawback in the use of DMARDs is their considerable toxicity [18].

The use of DMARDs for the management of periodontitis has been restricted largely due to the toxicity issues. However, the use of gold salts in an animal model has shown reduced periodontal destruction [19]. Till now, no human studies have been performed.

The relationship between rheumatoid arthritis (RA) and periodontitis is controversial. Many studies that have been done present conflicting results regarding the relationship between periodontitis and RA. However, a significant association between these two common chronic diseases has been reported recently [20,21].

RA is a common disease in Sudan, [22] and the literature correlating the severity of RA and the severity of periodontal disease is insufficient. This study was designed to investigate the periodontal status and in RA patients and to find if there is an association between RA and periodontal disease among patients in Khartoum State.

## Methods

Eighty Rheumatoid arthritis patients (RA) aged 20 -60 years old were examined at the common rheumatoid arthritis clinics in Khartoum State (University of Medical Sciences and Technology, Elribat University Hospital and Ibrahim Malik Teaching Hospital). All patients had intertmittently been taking various kinds of NSAIDs for long periods, some of them occasionally in conjunction with chloroquines. A group of eighty healthy individuals matched in age and gender was selected as a control group from co-patients and employees in the same centers.

### Inclusion criteria

#### Study group

- Patients (20-60 years old) diagnosed with RA with consideration of the disease duration.

- Willingness of the patient to participate in the study.

- Only partially (at least 8 teeth excluding 3^rd ^molar) or fully dentate patients were included in the study.

#### Control group

- Age group 20 - 60 years old

- Absence of rheumatoid arthritis, diabetes, hypertension and blood dyscriasis. This was determined by testing random blood sugar levels, measuring the blood pressure and doing a complete blood count for each patient prior to the examination.

- Willingness to participate in the study

- Only partially (at least eight teeth, excluding 3^rd ^molar) or fully dentate patients were selected in the control group.

Exclusion criteria included pregnancy, lactation, smoking, periodontal therapy or antibiotics in the previous three months, or any systemic condition which might have affected the progression of periodontitis. No subjects with localized or generalized aggressive periodontitis were included in this study.

Each subject who met the inclusion criteria completed a questionnaire, which gathered information on their demographic background. Approval for the research was obtained from the Research Ethics Committee of the Faculty of Dentistry University of Khartoum. Aims of the investigation and the nature of the study were fully explained to the subjects, who gave their informed written consent before participation.

All periodontal examinations were made with Michigan O periodontal probes with a controlled force of 0.2N by one examiner (Kamil S). Clinical measurements were made at four sites (mesiobuccal-distobuccal), (mesio lingual-distolingual) of all teeth, excluding third molars. Plaque Index (PI) [23] and Gingival Index (GI) [24] were used for detection of plaque and gingival inflammation respectively. Michigan O periodontal probe was used for measurement of pocket depth and clinical attachment level.

### Statistical analysis

Standard descriptive statistical techniques were used to summarize and present sample information. To check for possible significant differences in periodontal status between the case and control group, the t-test was used for normally distributed data. In case of non-normal data, the Mann-Whitney test was used. The data was processed using STATA software package (version10).

## Results

**Figure 1:**
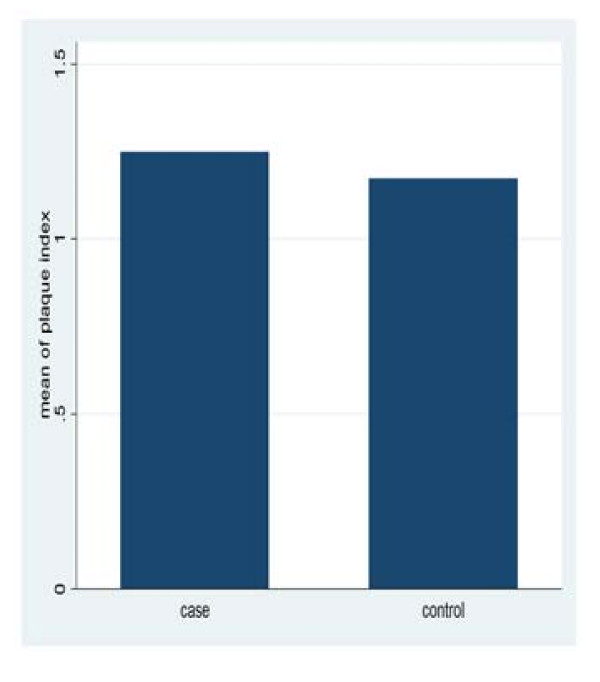
**Mean plaque index for study and control group**.

**Figure 2:**
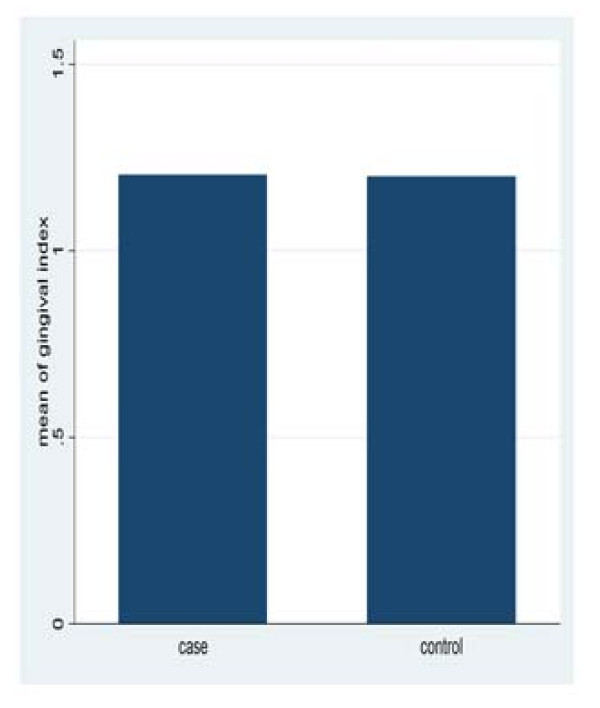
**Mean gingival index for study and control group**.

The data of this study was collected over a period of five months from three rheumatoid arthritis centers in Khartoum State. The results revealed that there were no significant differences in plaque and gingival index among study and control, with mean plaque index of (1.25 ± 0.4) for patients and (1.17 ± 0.28) for the control group (p = 0.3597). The mean gingival index was (1.2 ± 0.24) for the patients and (1.2 ± 0.33) for the control (p = 0.3049) (Figure 1, 2).

There was a statistically significant difference in the mean pocket depth between the study and the control group, a mean pocket depth of > 4 mm was observed in 10% of subjects in the RA group compared to 1.25% in the control group (table 1).

**Table 1:** Comparison of the probeable pocket depth (per millimeters) between study and control groups

	Cases (N = 80)	Controls (N = 80)	P Value*
≤ 0.5	52 (65%)	77 (96.25%)	0.000
> 0.5 to ≤ 1	20 (25%)	2 (2.5%)	
> 1 to ≤ 1.5	5 (6.25%)	1 (1.25%)	
> 1.5	3 (3.75%)	0 (0%)	

Mean ± SD	0.46 ± 0.42	0.15 ± 0.22	0.000

The results showed statistically significant differences in clinical attachment loss between study and control groups, with mean clinical attachment loss of (1.03 ± 0.95) for the study group and (0.56 ± 0.63) for the control group p = 0.0002 (table-2).

**Table 2:** Comparison of clinical attachment loss (per millimeters) between study group and control group

	Cases (N = 80)	Controls (N = 80)	P Value*
≤ 1.17	52 (65%)	70 (87.5%)	0.006
> 1.17 to ≤ 2.33	20 (25%)	7 (8.75%)	
> 2.33 to ≤ 3.5	5 (6.25%)	3 (3.75%)	
> 3.5	3 (3.75%)	0 (0%)	

Mean ± SD	1.03 ± 0.95	0.56 ± 0.62	0.0002

From the study, there was no significant correlation between duration of illness and plaque index (p = 0.9786), gingival index (p = 0.9079), and clinical attachment loss (p = 0.0933) at 0.05 level of significance (table-3).

**Table 3:** Association between RA drugs and periodontal parameters

	Plaque Index	Gingival Index	Probeable Pocket Depth	Clinical Attachment Depth
NSAIDs	NA	NA	NA	NA (0.674)
*P Value*	(0.718)	(0.343)	(0.228)	
Steroids	NA	NA	NA	NA (0.981)
*P Value*	(0.812)	(0.932)	(0.747)	
Pencilliamine	NA	NA	NA	NA (0.776)
*P Value*	(0.288)	(0.198)	(0.776)	
Methiotrxate	NA	NA	NA	NA (0.483)
*P Value*	(0.738)	(0.415)	(0.301)	
Hydroxychloroquirie	NA	NA	NA	NA (0.563)
*P Value*	(0.192)	(0.254)	(0.563)	
Sulphasalazine	NA	NA	NA	NA (0.590)
*P Value*	(0.944)	(0.569)	(0.837)	

NA no association

P-value is measured by chi-square test

The study also revealed that no association exists between the drugs used to treat rheumatoid arthritis (NSAIDs & DMARDs) and the periodontal parameters (plaque index, gingival index, probeable pocket depth and clinical attachment loss) (table-4).

**Table 4:** Relationship between duration of illness and periodontal parameters

	Plaque index	Gingival index	Pocket depth	Clinical attachment loss
**Duration of illness**	p-value (0.9786)	p-value (0.9079)	p-value (0.5978)	p-value (0.0933)

## Discussion

In this study, 72 females and 8 males participated (9:1). This ratio is greater than previous studies in other countries which reported that females were three times more likely to develop RA than males [25]. The present study reveals that there is no statistically significant difference in the plaque index between Ruematoid Arthritis patients and the control group (p = 0.524), and this is in agreement with Mercado F.B et al [21] and Yaniv et al [26]. Thus, the general concept that RA patients tend to have more plaque deposits because of limited dexterity was not validated. However Ishi et al [27] and Ezel et al [28] demonstrate a high prevalence of sites with dental plaque in their studies, and this may be explained by the fact that those patients directed their attention mainly to their serious illness while neglecting their oral health.

No significant difference has been found in the Gingival index when comparing the study group with the control group (p = 0.3049). This is similar to the findings reported by Mercado et al [21] and Ishi et al [27], but it disagrees with the findings by Yaniv et al [26] which found higher prevalence of sites with gingival inflammation in the RA patients compared with the control group.

There was a statistically significant difference in the mean pocket depth between the study and the control group. A mean pocket depth of > 4 mm was observed in 10% for the study group versus 1.25% for the control group, which indicated presence of deeper pocket depth in the RA patients. These results agreed with Mercado et al [21], Mikael et al [29], N pischon et al [8] and Yniv et al [26].

A significant difference has been observed in the clinical attachment level of study and control groups l (p = 0.002). The results agree with Ishi et al [27], Mikael et al [29] and Depablo et al, [30] and this may be due to increased secretion of pro-inflammatory mediators in both conditions.

Our findings showed that a relationship may exist between periodontitis and RA. This association is probably due to a common dysregulation of the immune-inflammatory response in these patients, despite their different etiology. In both conditions, there are a number of possible pathways of similar dysregulation, including characteristics of innate and acquired immune systems. Neutrophils play an important role in the pathogenesis of both diseases, and an aberrant neutrophilic response has been described, RA and periodontitis [31]. Another common pathogenic link affecting periodontitis and RA is the monocytic hypersecretory state [11], which may induce the secretion of excessive pro-inflammatory cytokine secretion, such as IL-1b, TNF-a and IL-6, which results in the stimulation of degrading enzymes and tissue destruction. The role of helper T lymphocytes has also been evaluated [32], it has been suggested that T-cell characteristics in periodontal diseases may resemble, in some respects, those in RA, a condition in which tissue destruction is mediated by a T helper 1 (Th1) cytokine profile. Some studies point to a genetic component in the susceptibility to RA and periodontitis. Associations with HLA subtypes and genes outside the HLA region, such as genetic polymorphisms of some cytokines, may contribute to susceptibility to both diseases [33,34].

This study shows no association between the drugs used for treatment of rheumatoid arthritis (NSAIDs & DMARDs) and the periodontal parameters (plaque index, gingival index, and clinical attachment loss). This agreed with findings of Gleissner et al [35], who found no correlation between the duration of pharmacotherapy and periodontal parameters, but it disagreed with Ezel et al [28] who found that medication including NSAIDs and corticosteroids may decrease gingival inflammation.

There was no significant association between the duration of illness and periodontal destruction, although many RA patients take medications that can reduce periodontal destruction (i.e., NSAIDs). This may indicate that prior to the development of RA symptoms, the periodontitis was most likely developing and not detected.

## Conclusion

The results indicated a significant relationship between periodontal disease and RA. However no significant difference between plaque and gingival index has been detected among study and control groups. This study highlights the potential for a relationship between two of the most common and debilitating chronic inflammatory conditions affecting the Sudanese population and warrants further detailed investigation.

## Abbreviations

RA: Rheumatoid Arthritis; NSAIDs: Non steroidal anti-inflammatory drugs; DMARDs: Disease Modifying Anti Rheumatoid Drugs; HLA: Human Leukocyte Antigen; PI: Plaque Index; GI: Gingival Index.

## Competing interests

The authors declare that they have no competing interests.

## Authors' contributions

SKA designed the study and carried out the data collection and the data analysis, BGG and EME supervised the research, NTH writing/editing of the article with assistant of BGG. All authors have read and approved the final manuscript.
